# A matter of timing

**DOI:** 10.7554/eLife.41523

**Published:** 2019-01-01

**Authors:** Michael W Perry, Claude Desplan

**Affiliations:** 1 Department of BiologyNew York UniversityNew YorkUnited States

**Keywords:** genetics, sexual differentiation, gene regulation, neurons, *C. elegans*

## Abstract

A genetic pathway that times development works together with the sex-determination pathway to control the timing of sexually dimorphic neural development in *C. elegans*.

**Related research article** Pereira L, Aeschimann F, Wang C, Lawson H, Serrano-Saiz E, Portman DS, Großhans H, Hobert O. 2019. Timing mechanism of sexually dimorphic nervous system differentiation. *eLife* **8**:e42078. doi: 10.7554/eLife.42078

The fate of a cell during development depends on several factors, such as its location in the body, developmental stage, and sex. However, most cells in the body do not ‘know’ what sex they are and function the same way, even though male and female cells have different sex chromosomes. How, then, do sexually dimorphic cells – the cells that are responsible for differences between males and females, such as differences in the adult brain – learn their sex during development?

The sex of an animal can modify the development of cells, such as neurons, in several ways. For example, males and females can produce the same type of cells, but then cause targeted cell death in a subset of cells in one of the sexes (Figure 1A; Kimura et al., 2008; Sanders and Arbeitman, 2008). Alternatively, an animal’s sex can modify the number of cell divisions, producing extra cells in one sex (Figure 1B; Emmons, 2018; Taylor and Truman, 1992; Sanders and Arbeitman, 2008). Or, cells that are identical at first can be modified in different ways in males and females (Figure 1C). This mechanism is common in the worm *Caenorhabditis elegans* (reviewed in Portman, 2017), but less common in insects like the fruit fly (see, for example, Kohl et al., 2013).

**Figure 1.**
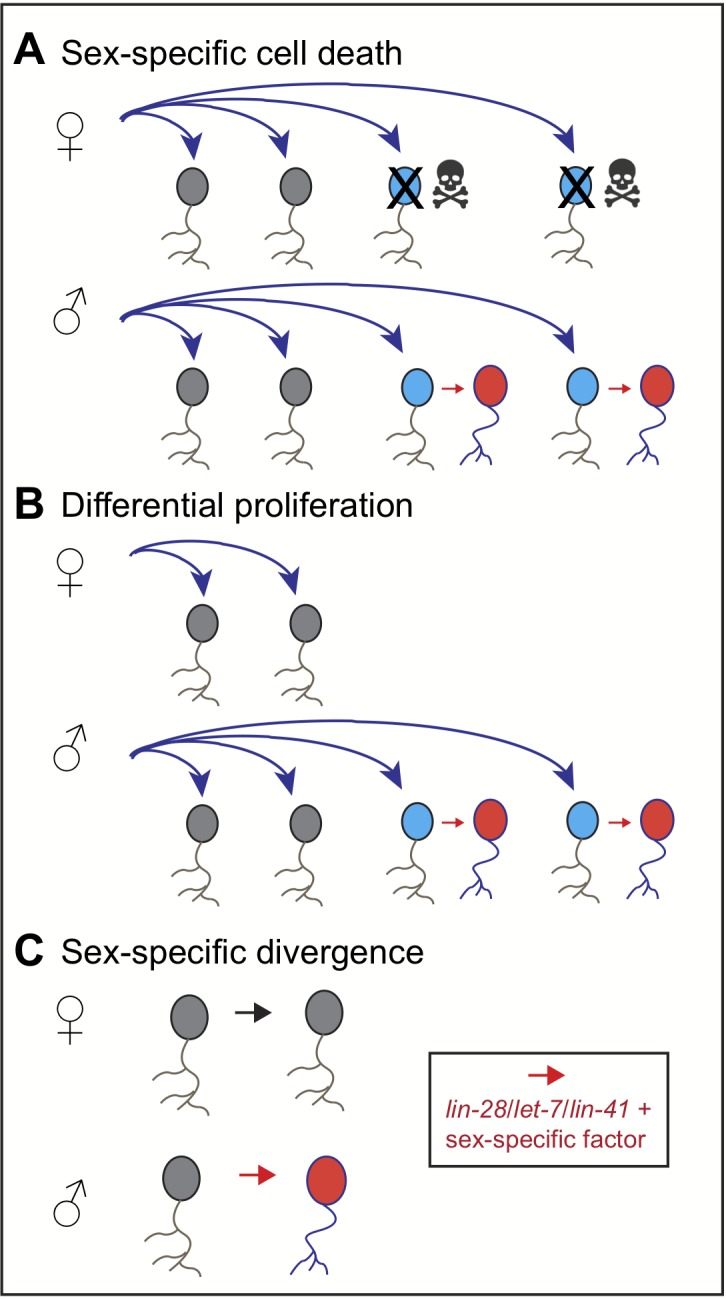
Sexual dimorphism in neurons. The sex of an animal can modify the development of sex-specific neurons in several ways. (**A**) Male and female animals may develop the same type of neurons (shown in grey), but over the course of development, one sex (females in this case) may lose some of the neurons (blue) due to programmed cell death (skulls). The neurons of the other sex (male neurons in this case) then acquire sex-specific features (red). (**B**) Input from the sex-specific pathway can modify the number of cell divisions, producing extra neurons (blue) in one sex, which then acquire their sex-specific features (red). (**C**) Alternatively, neurons can develop sex-specific features that affect their shape and/or properties (red). In *C. elegans*, this is mediated by combination of the heterochronic pathway (which involves the genes *let-7*, *lin-28* and *lin-4*1) and sex-specific genes, such as *lin29a* or homologs of *doublesex*.

In *C. elegans*, most of these differences arise during the fourth stage of larval development, just before they become sexually active, but how do cells know when to become different? Now, in eLife, Oliver Hobert of Columbia University and colleagues – including Laura Pereira as first author – report that the heterochronic pathway (which regulates the larval development of worms) is also involved in controlling the timing of sexual differentiation in the nervous system of *C. elegans* (Pereira et al., 2019).

Pereira et al. – who are based at Columbia, Rochester, and Basel – reveal that three genes (*let-7*, *lin-28* and *lin-41*) control when sexual maturation takes place in the neurons of *C. elegans.* Mutation of *let-7* precociously initiates sex-specific changes in neurons, while overexpression overrides these changes. Furthermore, in young worms, *lin-41* represses the production of a newly identified version of a gene called *lin-29a*, which is sexually specific. As the worms mature, however, the protein of *lin-28* is lost, which allows *let-7* to deactivate *lin-41*. In turn, *lin-29a* is then expressed in a subset of neurons. These neurons then turn on male-specific genes and adopt a male-specific shape and function (in the manner shown in Figure 1C). Both *let-7* and *lin-28* have been shown to control the timing of sexual differentiation in mice and humans, providing an intriguing hint of deeply conserved mechanisms (see, for example, Corre et al., 2016; Zhu et al., 2010; Chen et al., 2017).

The researchers discovered that the activation of *lin-29a* controls the sex-specific features of a neuron, called the AIM interneuron, which is important for the behavior of males. Male worms that lack this gene move in a way that is typical for hermaphrodites (modified females that can self-fertilize), providing a convincing demonstration that *lin-29a* provides the sex-specific input in the regulation of male genes. Pereira et al. further found that the pathway that determines the sex of a cell, including the gene *tra-1*, also regulates the production of *lin-29a*.

In other cells in *C. elegans*, homologs of a gene called *doublesex*, rather than *lin29a*, shape the sex-specific traits. The gene d*oublesex* is well-known for being involved in the sex-specific development of the nervous system in insects, although another gene called *fruitless* (which is not present in worms), is even more important in this process. Given the similarities, it is tempting to draw parallels between *lin-29a* and *fruitless*, though no direct homology has been identified.

It is unknown whether a heterochronic pathway similar to the one in *C. elegans* affects sex-specific cell fate decisions in fruit flies. The genes *fruitless* and *doublesex* only start to exhibit sex-specific expression in the nervous system at the end of the last larval stage and the early pupal stages (but as soon as the neurons have formed), which can then result in sexual dimorphism (as in Figure 1). This is different from what Pereira et al. report in *C. elegans*, where the pathway acts on many types of neurons at roughly the same stage, often well after they have been specified. Whether any neurons in *Drosophila* exhibit a similar delay in sex-specific fate specification after patterning is unknown.

The links to *let-7* and *lin-28* in the sexual differentiation of vertebrates raise the question of how precisely these genes are affecting their neural development during puberty; whether the number or types of neurons are modified by this pathway, as they are in *C. elegans*; and whether similar connections to homologs of *lin-29a* or *doublesex*-like genes exist. The study of Pereira et al. has set the stage for future work in flies and vertebrates to test potentially shared components and interactions.

## References

[bib1] Chen YC, Chen LM, Lin HH, Chen BH, Chao MC, Hsiao HP (2017). Association study of *LIN28B* in girls with precocious puberty. Journal of Pediatric Endocrinology and Metabolism.

[bib2] Corre C, Shinoda G, Zhu H, Cousminer DL, Crossman C, Bellissimo C, Goldenberg A, Daley GQ, Palmert MR (2016). Sex-specific regulation of weight and puberty by the *Lin28/let-7* axis. Journal of Endocrinology.

[bib3] Emmons SW (2018). Neural circuits of sexual behavior in *Caenorhabditis elegans*. Annual Review of Neuroscience.

[bib4] Kimura K, Hachiya T, Koganezawa M, Tazawa T, Yamamoto D (2008). Fruitless and Doublesex coordinate to generate male-specific neurons that can initiate courtship. Neuron.

[bib5] Kohl J, Ostrovsky AD, Frechter S, Jefferis GS (2013). A bidirectional circuit switch reroutes pheromone signals in male and female brains. Cell.

[bib6] Pereira L, Aeschimann F, Wang C, Lawson H, Serrano-Saiz E, Portman DS, Großhans H, Hobert O (2019). Timing mechanism of sexually dimorphic nervous system differentiation. eLife.

[bib7] Portman DS (2017). Sexual modulation of sex-shared neurons and circuits in *Caenorhabditis elegans*. Journal of Neuroscience Research.

[bib8] Sanders LE, Arbeitman MN (2008). Doublesex establishes sexual dimorphism in the *Drosophila* central nervous system in an isoform-dependent manner by directing cell number. Developmental Biology.

[bib9] Taylor BJ, Truman JW (1992). Commitment of abdominal neuroblasts in *Drosophila* to a male or female fate is dependent on genes of the sex-determining hierarchy. Development.

[bib10] Zhu H, Shah S, Shyh-Chang N, Shinoda G, Einhorn WS, Viswanathan SR, Takeuchi A, Grasemann C, Rinn JL, Lopez MF, Hirschhorn JN, Palmert MR, Daley GQ (2010). *Lin28a* transgenic mice manifest size and puberty phenotypes identified in human genetic association studies. Nature Genetics.

